# Occurrence and seasonal disparity of emerging endocrine disrupting chemicals in a drinking water supply system and associated health risk

**DOI:** 10.1038/s41598-022-13489-3

**Published:** 2022-06-03

**Authors:** Manoj Kumawat, Poonam Sharma, Namrata Pal, Meenu Mariya James, Vinod Verma, Rajnarayan R. Tiwari, Swasti Shubham, Devojit Kumar Sarma, Manoj Kumar

**Affiliations:** 1 ICMR-National Institute for Research in Environmental Health, Bhopal Bypass Road, Bhouri, Bhopal, Madhya Pradesh 462030 India; 2 Department of Hematology, Stem Cell Research Centre, Sanjay Gandhi Post-Graduate Institute of Medical Sciences, Lucknow, Uttar Pradesh 226014 India

**Keywords:** Environmental sciences, Risk factors

## Abstract

Contamination of drinking water with endocrine-disrupting chemicals (EDCs) raises concerns over the security and long-term sustainability of clean water supplies as well as human exposure via daily water intake. In this study, the seasonal disparity and occurrence of six phthalates and bisphenol-A in the drinking water supply system and associated health-risk were examined. The detection frequencies of the ∑6PAEs ranged from 24 to 100% in the winter whereas; in summer it is below the detection limit up to 100%. DEHP was the most prevalent phthalate congener ranging from 1.14 to 8351.85 µg/L (winter) and 0.552 to 410.29 µg/L (summer) surpassing the permissible limit. However, BPA concentrations were found under the permissible limit. The results suggested that PAEs concentration displayed significant seasonal variations with the highest in winter and the lowest in summer. The exposure to PAEs and BPA from drinking water was assessed, indicating a possible health risk to humans with a hazard quotient (HQ) > 1 for DEHP only. The findings necessitate an immediate scrutiny of these EDCs in drinking water supply system and are critical for implementing effective technologies at the WTP scale to ensure the quality and safety of drinking water to ascertain human and environmental health.

## Introduction

The safety and purity of drinking water are critical to human health across the world. As a result of uncontrolled anthropogenic activity, surface water is exposed to a myriad of contaminants, making clean drinking water a primary focus. Contamination of drinking water with EDCs such as phthalate esters (PAEs) and bisphenol-A (BPA) has physiological consequences by simulating hormones or interfering with their production, release, and metabolic pathways. Low molecular weight phthalates like dimethyl phthalate (DMP), diethyl phthalate (DEP) and benzyl butyl phthalate (BBP) are challenging chemicals as they mimic endocrine hormones and play a role in several non-communicable diseases like infertility, diabetes, obesity, neurological diseases, and aberrant growth patterns^1^. Whereas, high molecular weight phthalates such as di (2-ethylhexyl) phthalate (DEHP), di-*n*-butyl phthalate (DBP), and di-n-octyl phthalate (DNOP) are suspected carcinogens, that could harm the liver, excretory system and reproductive system. Many PAEs have teratogenic and mutagenic potency, while some of the PAEs also exhibit estrogenic activity. Of all known PAE congeners, DMP, DEP, DEHP, DBP, DNOP and BBP were identified as priority pollutants due to their targeted influence on the environment and human health^2,3^.

PAEs are semi-volatile, polar organic molecules, with high solubility^4^. These plasticizers are not firmly attached to the matrix, even minor changes in environment conditions (such as temperature, pH, pressure and presence of solvent and additives) and physiochemical parameters of PAEs (i.e. solubility, vapor pressure, and diffusion coefficient) might cause them to migrate to the surroundings^5^. Around 6 million tons of phthalates are manufactured annually across the world^2^. They are widely used in a variety of industries including construction, pharmaceuticals, food packaging, and medical equipment^6^. DEHP being the most common plasticizers in the world is extensively used in the synthesis of plastic materials such as polyvinyl chloride (PVC), and in the manufacturing of rubber, styrene, lubricants, glues, paints, pharmaceutics, cosmetics and pesticides. BBP is found in polyurethane, polysulfide and acrylic-based polymers whereas DBP is used as a plasticizer in PVC and rubber as well as in paints and cosmetics as a solvent and fixative. DNOP is widely used in floors, coverings, conveyor belts and other applications. Furthermore, PAEs like DMP and DEP have a wide range of uses in cosmetics and baby care products manufacturing^7^. The presence of PAEs such as DMP, DEP, DEHP, DBP, DNOP, and BBP has been detected in drinking water, raising serious concerns about human health^8,9^. The threshold value for DEHP in drinking water (category B, includes probable human carcinogens) has been set to 6 µg/L and 8 µg/L by EPA and WHO respectively^10–12^.

BPA is primarily employed as a monomer in the manufacturing of flame retardants polycarbonate, epoxy and unsaturated polyester-styrene resins. The resulting materials are frequently utilized as can coatings, powder paints, dental fillings, and household items such as bottles, cutlery and containers. BPA is currently produced at a rate of about 8 million tonnes per year, with the international market expected to remain stable at around 7300 k tonnes by 2023^13^. BPA-containing goods are officially prohibited in some American states and Canada due to their endocrine disruptive properties, yet they are nevertheless widely used in other nations^14^. It can be released into the surrounding environment during commercial processing and as leachates from the finished products. One of the important sources of human exposure to BPA is drinking water and might be associated with a number of chronic human disorders including cardiovascular disease (CVD), birth abnormalities, respiratory and kidney problems and breast cancer according to epidemiological research. It has also been found to have estrogenic activity, which could increase the risk of cancer by interrupting the developmental process^15,16^.

In most of the countries, natural reservoirs such as rivers, lakes, dams, etc. are the primary source for drinking water treatment plant (DWTP). Direct sources, such as untreated and treated discharges and indirect sources such as runoff and leachates are the two main causes of EDCs contamination in surface water. The DWTP is the last stage of EDCs exposure between the drinking water supply and the consumers. However, standard drinking water treatment facilities are insufficient for eliminating these pollutants, resulting in their dissemination into the drinking water supply^17^. Despite of the extensive use and prevalence of plasticizers in the environment, developing countries like India still lack the awareness and strict guidelines on environmental monitoring of these pollutants. In India, dams, rivers, and lakes are popular source of drinking water that often receives untreated industrial effluents. Moreover, there is no baseline data available for India regarding the presence or quantity of PAEs and BPA in drinking water. Hence, this study has been carried out to detect and quantify the EDCs in drinking water system and their associated health risks.

The present study was undertaken to quantify selected EDCs i.e., PAEs and BPA across the drinking water supply system, evaluate their potential influence on drinking water security and assess their health risk. The hazard quotient (HQ) was used to estimate the human health risk caused by the targeted EDCs in the drinking water. The seasonal distribution pattern of these pollutants was also investigated in order to establish the correlation between the abundance of phthalate esters and BPA with that of temperature variation.

## Material and methods

### Study area and sample collection

**Figure 1:**
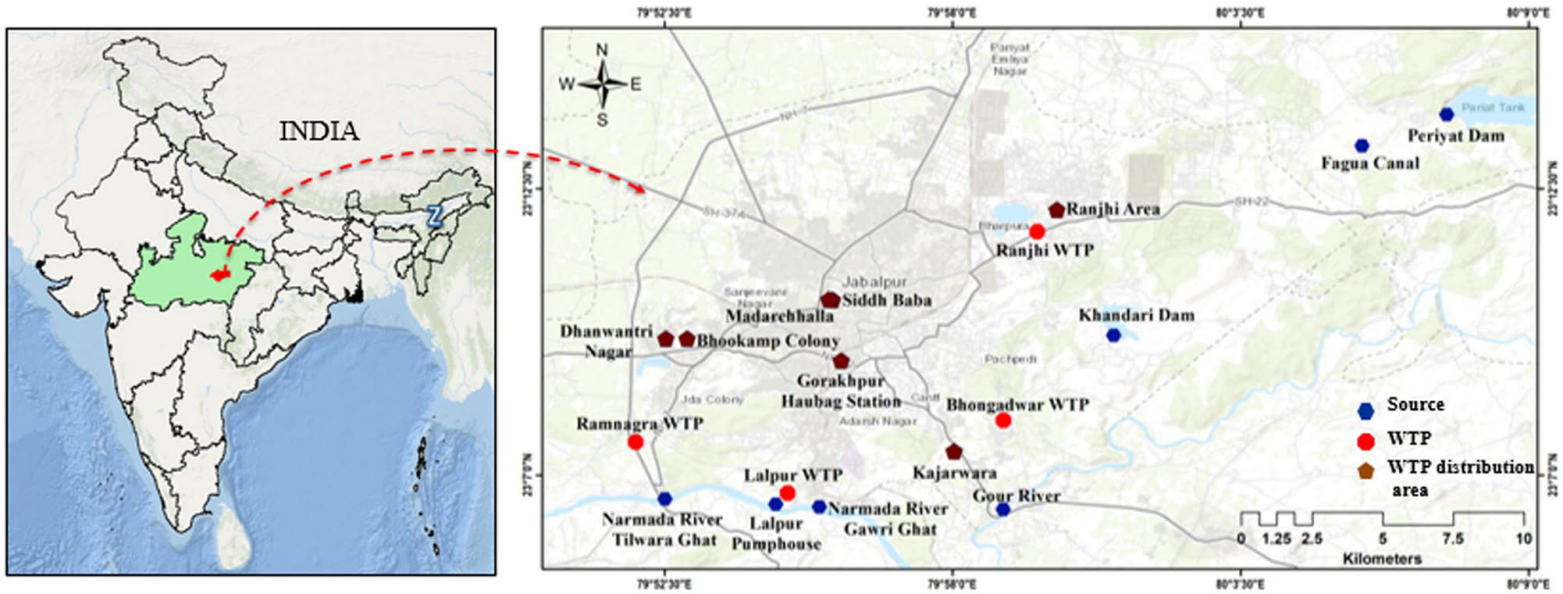
GIS Map illustrates the sampling sites of the Jabalpur city. Surface: River and Dam; WTP; WTP distribution area: includes residential area from where OH and DW samples were collected. Map was created using ArcGIS (Desktop Version 10.8.1), https://www.esri.com/en-us/arcgis/products/arcgis-desktop/overview.

The study was conducted in Jabalpur city of Madhya Pradesh, India. The sampling area covers all community water sources (surface water) like rivers (Narmada and Gaur), reservoirs and dams and the public water supply systems. Four WTPs at different locations, namely Ranjhi, Bhongadwar, Lalpur and Ramnagra were chosen for the study showed in Fig. 1, created by the Arc GIS Desktop 10.8.1 software. Samples were collected from the surface as well as distribution areas of these WTPs and also the WTP itself for a comprehensive assessment of EDCs in the water supply chain. In each WTP distribution area, water samples were collected from overhead tank (water storage tank constructed over individual houses for domestic purpose) and drinking water system (Filtration unit/tap water) of 25 household. Assessment of EDCs at each stage is crucial to check the abundance pattern of these contaminants across the water supply line as environmental factors and leaching activity may vary at each stage. In the present study, the samples were collected during both the winter season (Jan-2021) and summer season (June-2021) to study the seasonal variations. Samples were collected in pre-cleaned 1 L amber glass bottles, using phthalate-free caps and were preserved with 80 mg/L of sodium thiosulfate to quench residual chlorine in the finished water^18^. The samples were packed and transported in cold condition to the laboratory overnight and stored in the dark at 4 °C until extraction. The surface (influent for drinking water treatment plants) and WTP effluent (i.e., finished drinking water ready for distribution) samples were collected on the same day with the assumption that the source water characteristics would not vary significantly throughout the day.

### Chemicals and reagents

A standard stock solution containing phthalate esters mixture(606-M) of dimethyl phthalate (DMP), diethyl phthalate (DEP), di-butyl phthalate (DBP), butyl benzyl phthalate (BBP), bis (2-ethylhexyl) phthalate (DEHP), di-*n*-octyl phthalate (DNOP) (each at a concentration of 0.2 mg/mL in methanol) and bisphenol-A (10 mg/mL) were purchased from AccuStandard Chem. Co. (CT, USA). HPLC grade acetone, methanol, dichloromethane and sodium chloride (99.5%) was obtained from Merck (Darmstadt, Germany). Working standard solutions for calibration were prepared from the stock standard solutions and stored at 4 °C.

### Sample extraction and analysis

Extraction of phthalates and BPA were done via salt-assisted liquid–liquid extraction (SALLE) as described in EPA (Method 3510)^18^. Water-immiscible solvents, like dichloromethane (DCM) and *n*-hexane, were tested to extract and pre-concentrate the PAEs and BPA from the water samples. DCM was found to be the suitable solvent for selective extraction of all the targeted six PAEs and BPA with maximum recovery in comparison to *n*-hexane. A serial liquid–liquid (LLE) extraction was performed in a 2 L separatory funnel, with 1 L of the sample (containing 990 mL water sample, 10 mL 0.5 M phosphate buffer (pH = 7), and 50 g NaCl) and was extracted out thrice with 60 mL DCM each time. After each extraction, the organic layer was allowed to separate for 10 min. The pooled extracts were evaporated on a rotary evaporator (45 °C and 60 rpm). The dried extracts were dissolved in 5 mL of GC/MS grade methanol and concentrated up to 1 mL under a stream of N_2_ at 65 °C. This eluate was transferred to GC vials and used for the GC–MS analysis. To check the extraction efficiency, milli-Q water was spiked with known concentrations of PAEs and BPA and extracted using the same method.

**Figure 2:**
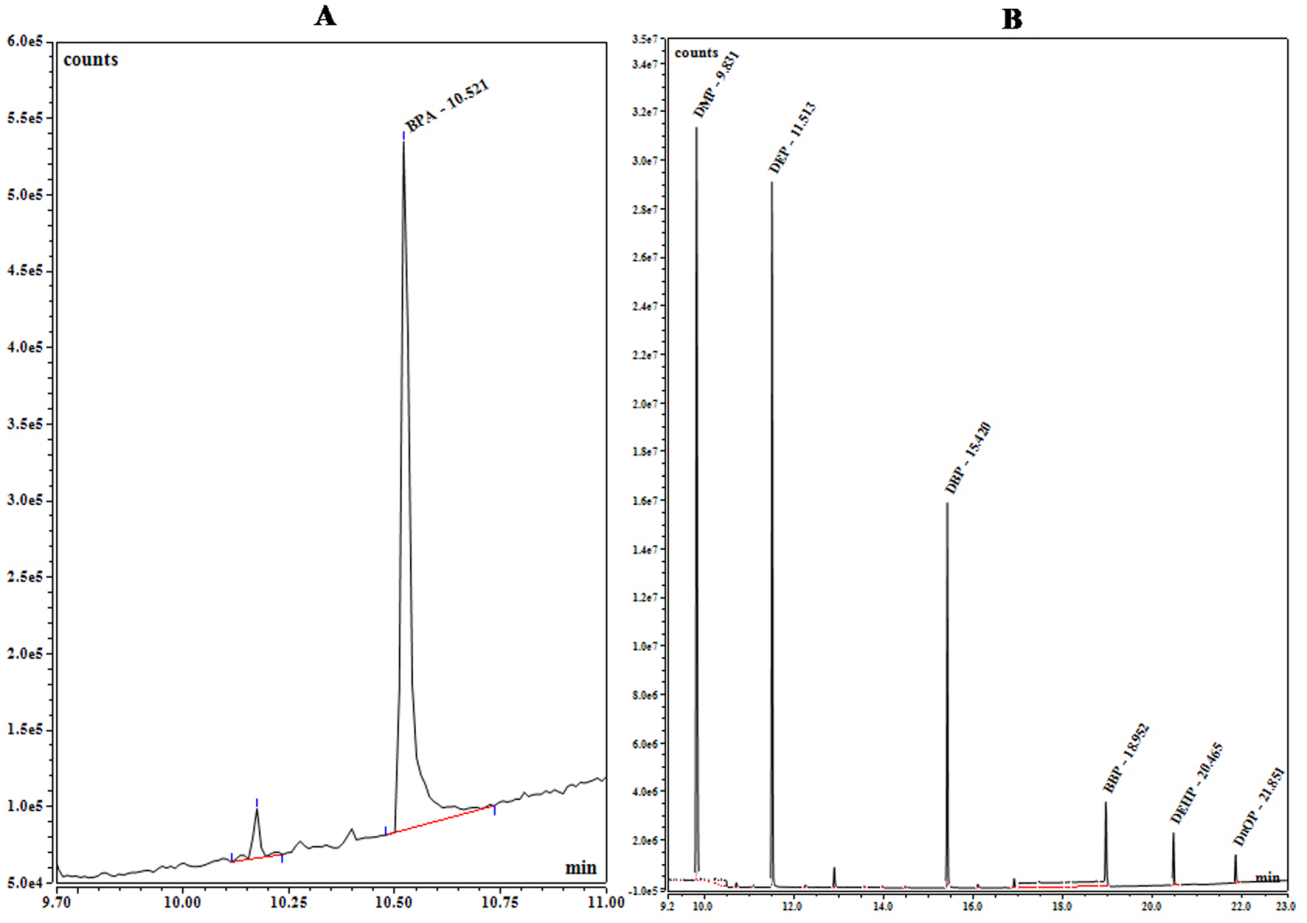
Ion Chromatogram of (**A**) BPA (Bisphenol-A) and (**B**) PAEs Congeners and their peak identities: *DMP* Dimethyl phthalate, *DEP* Di-ethyl phthalate, *DBP* Di-*n*-butyl phthalate, *BBP* Benzyl butyl phthalate, *DEHP* Di (2-ethylhexyl) phthalate, and *DNOP* Di-*n*-octyl phthalate were generated by using Chromeleon Console software (Version 7.2.10).

Gas chromatography (Thermo fisher Trace 1300 model) coupled with a mass spectroscopy ISQ 7000 (Thermo fischer, Italy) was used for the quantification of phthalates and BPA. Data acquisition and processing were performed using Chromeleon Console software (Version 7.2.10) https://www.thermofisher.com/order/catalog/product/CHROMELEON7. A TG-5MS Trace (30 m × 0.25 mm × 0.25 μm) fused silica capillary column was used. The flow rate of the carrier gas He (99.999% purity) was kept constant at 1.5 mL/min. The sample (1 μL) was injected into GC in split-less mode with an inlet temperature of 280 °C. Initially, the column temperature was programmed at 80 °C for 1 min and then elevated to 290 °C at the rate of 10 °C/min with a holding time of 8 min for PAEs. For BPA an initial temperature of 100 °C was provided for 2 min then increased to 290 °C at the rate of 20 °C/min with a holding time of 8 min. The mass spectrometer was operated in electron ionization (EI) mode at 70 eV and an emission current of 60 μA. Detector temperature was maintained at 280 °C. MS was operated in full-scan mode from *m/z* 40 to 400 for qualitative analysis. Acquisition for quantitative analysis was performed in the single-ion monitoring (SIM) mode along with characteristic ions (Table 1) and quantification was done with an external calibration method^19^. In order to prevent the background contamination of PAEs and BPA, all the employed glassware and apparatus were cleaned via analytical grade acetone and heated at 250 °C for 2 h. The limit of detection (LOD) and limit of quantification (LOQ) for all the six phthalates and BPA were calculated based on a signal-to-noise ratio of 3 and 10 times respectively. Details of quality assurance for phthalates and BPA analysis, including the limit of detection (LOD), the limit of quantification (LOQ), R^2^, and precision values (% RSD) are given in Table 1. During the GC–MS analysis, for every set of 10 samples, a set of blanks and standards were also run to ensure the performance of the instrument. The ion chromatograms of PAEs and BPA were obtained using GC/MS depicted in Fig. 2. The calibration curves for each targeted phthalate and BPA are provided in the supplementary file (Supplementary Fig. 1).

**Table 1:** QA/QC parameters for phthalates and BPA extraction and analysis.

Analytes	MW	CAS number	Retention time (min)	Monitored ions (*m/z*)	LOD (µg/L)	LOQ (µg/L)	R^2^	Precision (% RSD)
Dimethyl phthalate	194	131-11-3	9.8	163, 77	0.002	0.006	0.999	2.01
Diethyl phthalate	222	84-66-2	11.5	149, 177	0.0034	0.01	0.997	1.57
Di-*n*-butyl phthalate	278	84-74-2	15.4	149	0.01	0.03	0.995	1.80
Benzyl butyl phthalate	312	85-68-7	18.95	149, 206, 91	0.006	0.02	0.998	6.17
Bis(2-Ethylhexyl) phthalate	390	117-81-7	20.4	149, 167, 279	0.008	0.026	0.995	2.61
Di-*n*-octyl phthalate	390	117-84-0	21.8	149, 279	0.006	0.018	0.998	5.49
Bisphenol A	228	80-05-7	10.5	213, 119, 228	0.02	0.086	0.986	1.66

### Human health risk assessment

To validate the safety of drinking water from the viewpoint of carcinogenic and endocrine-disrupting potential, daily intake of targeted analytes were estimated based on their maximum concentrations detected in drinking water. By using human risks assessment, we can easily predict the possibility of health effects in the study population exposed to such EDCs. The health risks associated with EDCs are divided into carcinogenic and non-carcinogenic risk. In this study, we estimated carcinogenic as well as non-carcinogenic risks. Calculation was done on the basis of the toxicity data available in the Integrated Risk Information System (IRIS) of the Environmental Protection Agency (EPA) and World Health Organization (WHO).

The exposure levels and risk of phthalates and BPA for adults were calculated by using the following formula^20^.

$$ {\text{EDI}} = \frac{{{\text{MC}} \times {\text{IR}}}}{{{\text{BW}}}}, $$

where, EDI represents estimated daily intake (EDI) of PAEs and BPA, MC represents maximum concentration of the analyte, IR represents ingestion rate of drinking water (assumed as 4.0 L/day) per kg body weight (BW) of an adult. The calculated value is expressed in terms of ug/kg body weight/day^21^.

Non-carcinogenic risks induced by PAEs and BPA on human health were estimated using hazard Quotient (HQ). HQ was estimated by the following equation

$$ {\text{HQ}} = \frac{{{\text{EDI}}}}{{{\text{RfD}}}}, $$

where, RfD is oral reference dose in mg/kg body weight/day. It is an estimate of a daily exposure to human population.

For the estimation anti-androgenicity risk, HQ-AA was calculated using oral reference dose (RfD for anti-androgenicity)^22^. If HQ and HQ-AA > 1, then it indicates potential human health risk and vice versa.

Additionally, the contribution of the daily intake of DBP, BBP, and DEHP via consumption of drinking water was computed as:

$$ {\text{Contribution}}\,{\text{by}}\,{\text{drinking}}\,{\text{water}} = \left( {\frac{{{\text{EDI}}}}{{{\text{TDI}}}}} \right) \times 100, $$

where, TDI is the tolerable daily intake, defined as the estimated amount of a substance in drinking water, expressed on a bodyweight basis (mg/kg of body weight), that can be ingested over a lifetime without appreciable health risk^11^.

Carcinogenic risk through the oral route was estimated as excess cancer risk as follows:

$$ {\text{Excess}}\,{\text{Cancer}}\,{\text{Risk}} = {\text{CSF}} \times {\text{EDI,}} $$

where, CSF is the slope factor used to determine the cancer risk associated with the oral exposure of either carcinogen or probable human carcinogen. The CSF value for DEHP is 1.4 × 10^–2^ per mg/kg/day. The acceptable range for the assessment of excess cancer risk is 10^–5^ to 10^–6^, as per US EPA^10^.

### Statistical analysis

All statistical analyses were conducted using R version 4.1.0 using the packages ggplot2 (version 3.3.5), ggpubr (version 0.4.0) and dplyr (version 1.0.7). As per the requirement for the data analysis, descriptive analyses such as median and interquartile range (IQR) were calculated. Shapiro–Wilk test was performed to check the normality of the data. The scores were found to be significantly deviating from the normal distribution at a *p* ≤ 0.05 significance level. Therefore, Wilcoxon signed-rank test, a non-parametric statistical hypothesis test was used for comparing the PAE and BPA concentrations across the household water samples to assess whether their population median rank differs. A *p* ≤ 0.05 was considered to be statistically significant throughout the analysis.

## Results and discussion

### Occurrence and seasonal distribution of ∑6PAEs and BPA in the drinking water supply system

Among the seven analyzed molecules, the detection frequencies (DF) of the six PAEs ranged from 24 to 100% in the winter season whereas; in summer it is below the detection level in case of BBP to 100% (DEP, DBP, DEHP) in the sampling areas. Detailed information about the values of ∑6PAEs and BPA are summarized in Supplementary Table 1. In a study conducted by Luo et al.^23^, PAEs detection frequencies ranged from 1.8 to 81.8%. According to our results, DEHP was the major phthalate with the highest detection frequency of 100% (Supplementary Table 1), which is consistent with previous findings that reported its highest detection rate with 61%^24^, 93.3%^25^ and 92%^26^, indicating the ubiquity of DEHP in source and drinking water samples. The detection frequency for BPA is 100% in summer season whereas in winter it lies in the range of 64–100% as presented in Supplementary Table 1. A similar DF (67.6%) was reported for the surface water by Lalwani et al.^27^. The earlier recorded detection frequencies for BPA in drinking water were 5% (North America), 52% (Europe), and 59% (Asia) whereas for surface water it was reported 17%^28^.

**Figure 3:**
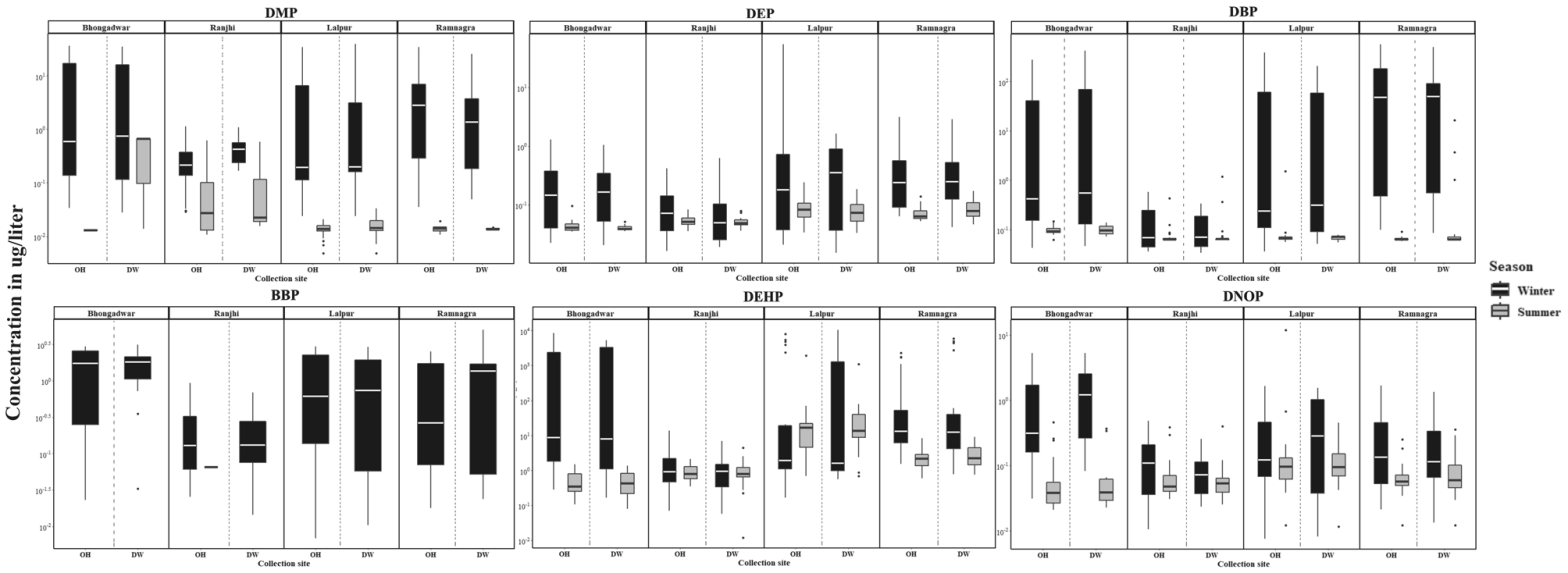
Seasonal comparison (**A**) winter and (**B**) summer, of dimethyl phthalate (DMP), diethyl phthalate (DEP), Di-*n*-butyl phthalate (DBP), Benzyl butyl phthalate (BBP), di (2-ethylhexyl) phthalate (DEHP) and Di-*n*-octyl phthalate (DNOP) in different study areas.

**Figure 4:**
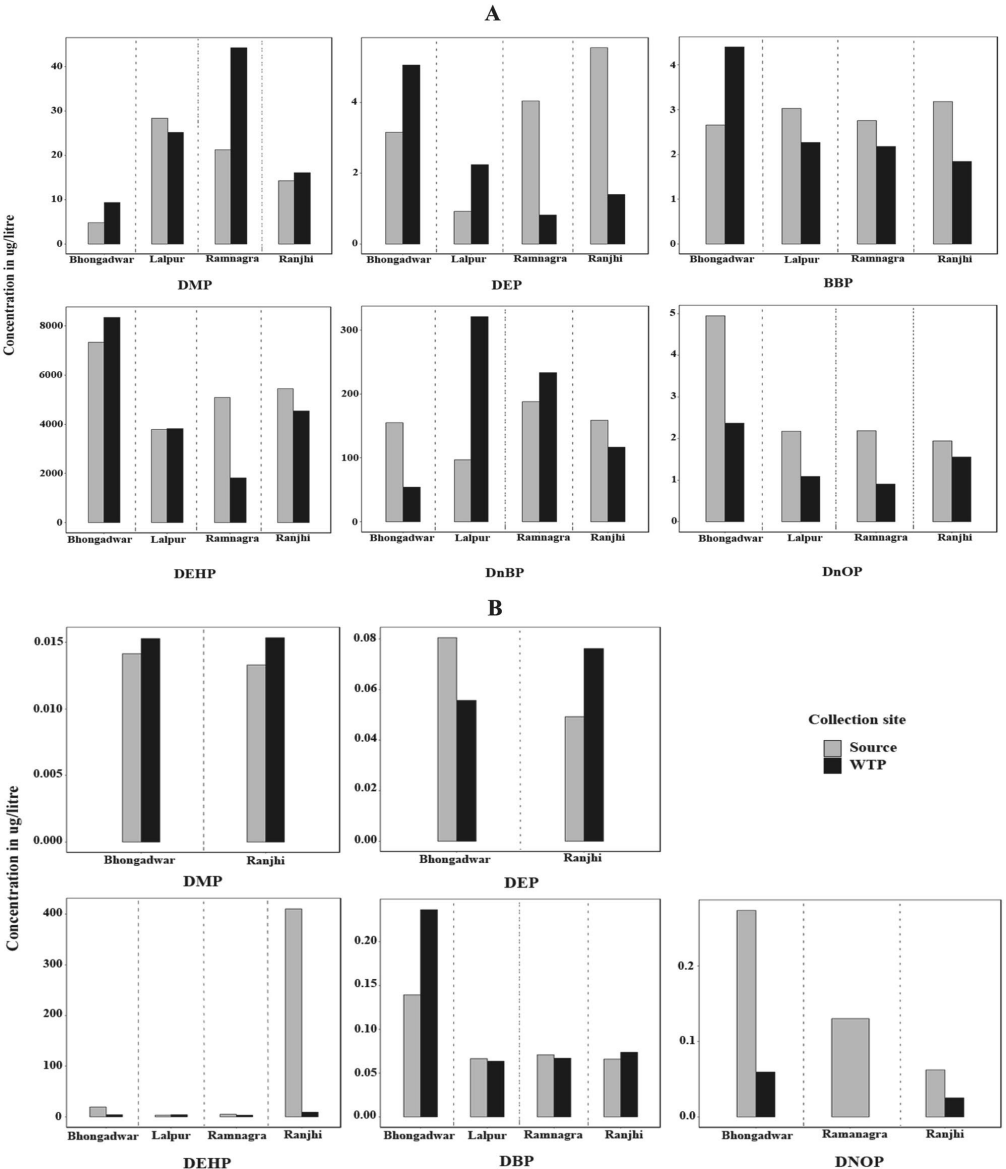
Area wise concentration of phthalates (µg/l) in Overhead and Drinking water during winter and summer season. Box whisker plots showing the lower quartile, mean, upper quartile ranges of concentrations of dimethyl phthalate (DMP), diethyl phthalate (DEP), Di-*n*-butyl phthalate (DBP), Benzyl butyl phthalate (BBP), di(2-ethylhexyl) phthalate (DEHP) and Di-*n*-octyl phthalate (DNOP) in Overhead (OH) and Drinking water (DW) samples over two seasons. BBP was detected only in one sample during summer thus not shown in the above figure.

The relative abundance of different PAE congeners in source water varied in the order DEHP > DBP > DMP > DEP > DNOP > BBP and DEHP > DNOP > DBP > DEP > BBP > DMP for winter and summer, respectively. The area-wise concentration of each PAE in surface, WTP, and drinking water samples (OH and DW) during winter and summer seasons are compared in Figs. 3, 4. The concentrations of PAEs (Σ6PAEs) lies in the range of 3927.89–7553.94 µg/L and 4.665–410.48 µg/L (surface water), 4173.91–8633.57 µg/L and 3.31–9.81 µg/L(WTP), 3.02–1527.29 µg/L and 0.722–90.47 µg/L (overhead water), 1.86–1438.20 µg/L and 0.77–57.75 µg/L (drinking water), for winter and summer respectively. Significant seasonal variations were observed for all PAE congeners, with a higher concentration of total PAEs detected in the winter season than in summer. Overall, the PAE concentrations in source water samples were found to be more than WTP effluents and drinking water samples. The results of this study suggest that WTP might be able to remove phthalates to some extent, but still, a substantial quantity of phthalates has been detected in drinking water.

Five phthalates such as DMP, DEP, DEHP, DBP and DNOP were frequently detected in all the samples except BBP (detected in summer only); of all, DEHP was consistently high in terms of concentration and occurrence during the two seasons. A significant seasonal variation was observed in five of the PAEs in the household over-head (OH) tank water samples and drinking water (DW) samples. The concentration of different PAEs was observed to be significantly varying with the season for the source water samples (Supplementary Table 2).

Considering the concentration values, DEHP, DBP and DNOP were the prominent congeners in the sampling area. Huike et al*.* reported the similar trend in the surface water from the Pearl River^29^. The abundance of DEHP, DBP, and DNOP in the surface water could be linked to their huge production and rampant usage. DEHP was the most prominent species in the water samples with a concentration range of 84.49–8351.85 µg/L, which is much higher than the earlier reported concentration of 1.28–5.28 µg/L^30^ and 1.684 µg/L^12^ in drinking water. Many previous studies have also reported similar results^9,23,31^. The reported DEHP concentrations were up to 2.6 µg/L and 27.1 µg/L in the treated wastewater^32,33^ and 490 mg/kg dry weight in the sludge^34^. Previously conducted study by Cruz-lopez et al*.* showed DEHP concentration range between 1 and 220 µg/L in surface water and 180–220 µg/L in ground water^35^. A high concentration of DEHP in source water may indicate its stronger sorption capacity and lower degradation rate. Moreover, the abundance of DEHP in the surface waters could be attributed to industrial effluents which are routinely discharged into rivers. Furthermore, the predominance of DEHP in the air may also contribute to the profusion of PAEs in source water^31^, and being a long alkyl chain PAE its mineralization is slow^36^.

Toxicological data on DNOP are extremely limited to date; therefore, its health and environmental risks are still ambiguous. Distribution of DMP and DEP in terms of concentration and abundance were found to be least of all the detected PAE congeners in source and drinking water, similar to those observed in the Jiulong River estuary, Southeast China^37^. The measured Σ6PAE concentrations unveiled notable seasonal variations with the highest value in winter and the lowest value in summer, which are consistent with the previous observation^38^. The lower concentrations of Σ6PAE in the summer could be partly due to high photolytic activity, microbial degradation, and oxidation^39–41^. Additionally, PAEs concentration in the air surrounding the source water might vary over seasons and could be one of the contributing factors for seasonal variations in PAEs levels. A previous study from Tianjin, China by Kong et al*.* reported that high concentration of phthalates was observed during winter than those in spring and summer in urban sites^42^. Besides this, a shift in the microbial communities and their relative abundance were subject to the seasonal variation that could change the rate of microbial mediated degradation.

The ongoing trend of phthalate contamination in drinking water calls for scrutiny as chronic/long-term exposure to PAEs has been linked to several health-related ailments including endocrine system disruption, metabolic diseases, cancer, developmental aberrations etc.^43^. In addition, implementation of upgraded technologies in WTPs that can remove phthalates more efficiently during the water treatment process may be considered.

**Figure 5:**
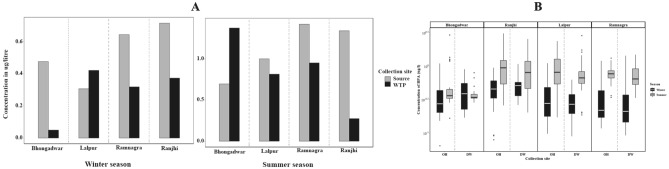
(**A**) Area wise and seasonal comparison of Bisphenol A (BPA) concentrations in source and WTP water samples, (**B**) Area wise concentration of Bisphenol A (µg/L) in Overhead and Drinking water during winter and summer season. Box whisker plots showing the lower quartile, mean, upper quartile ranges of concentrations of BPA in Overhead (OH) and Drinking water (DW) samples over two seasons.

Area wise BPA concentrations in source and drinking water were summarized in Fig. 5. Its concentration ranges 0.307–0.726 µg/L and 0.884–1.42 µg/L in surface water, 0.05–0.422 µg/L and 0.812–1.373 µg/L in WTP, 0.06–1.188 µg/L and 0.166–3.059 µg/L in OH water, 0.002–1.418 µg/L and 0.185–2.843 µg/L in DW for winter and summer, respectively (Fig. 5). From the four study areas, only OH water samples collected from Bhongadwar showed a statistically significant seasonal variation (*p* = 0.01755), whereas no significant seasonal difference was observed in any of the other study areas, for both the OH and DW samples (Supplementary Table 2). An earlier study has reported a much higher concentration of BPA i.e., 85.5 µg/L and 2.230 µg/L in surface and drinking water, respectively^44^. The Study conducted in Mexico, evaluated BPA concentration upto 30 µg/L (surface water) and 25 µg/L (ground water)^35^. In the summer season, the BPA concentrations in the water samples were generally high and it seemed to be well distributed in terms of occurrence which was consistent with the results reported by Ref.^45^ while Luo et al*.* observed the opposite trend. The high abundance of BPA in summer could be related to the temperature, as it gets leached out under high temperature or acidic/alkaline environments^46,47^. The concentration of BPA in our study was higher than that observed in three rivers (Kaveri, range 6.6–136 ng/L; Vellar, range 2.8–6 ng/L and Tamiraparani, range 9.8–36 ng/L) in Southern India^26^. Furthermore, Arnold et al*.* reported 0.099 µg/L, 0.014 µg/L, and 0.317 µg/L concentrations for North America, Europe, and Asia, respectively^28^.

Among the phenolic compounds, BPA is the most reported chemical in surface water around the world^48–50^. The average concentration of BPA in the Ganga River was found to be 0.04–4.46 µg/L^51^. Lalwani et al*.* reported 1.42 µg/L and 14.8 µg/L concentrations in the Cooum River and Yamuna River of India, respectively^27^. Among the reported data, the highest BPA concentration was observed in the Yamuna River of India, till date. Though BPA was found in source and drinking water, the mean concentrations were lower than those of PAEs irrespective of the seasons and detected concentration lies within the admissible limit. The source water is usually chlorinated in WTP; it may also affect the BPA concentration in the drinking water supply as it degrades quickly in highly chlorinated water^48^. Furthermore, an earlier study had shown the abiotic process mediated transformation and mineralization of BPA under anoxygenic conditions, suggesting that it is susceptible to degradation under such conditions^52^. Therefore, it can be inferred that the seasonal fluctuations (due to weather conditions like temperature, intensity of sunlight, rainfall etc.) have a strong relationship with that of EDCs concentration and their distribution in source and drinking water^46^.

### Human health-risk

We calculated the daily consumption of PAE congeners and BPA based on the maximum concentration in drinking water. The hazard quotient (HQ and HQ-AA) associated with PAEs and BPA exposures in different seasons are summarized in Table 2. Of all, DEHP has a higher (> 1.0) HQ and HQ-AA irrespective of the season, showing a higher risk for human health. This result is in contrast to the study conducted by Zare et al*.* which showed that HQ-AA for DEHP is less than 1.0 in Iran^22^ In a similar line, Abtahi et al*.* reported a much lower HQ for DEHP in Tehran^53^. The exposure of other targeted PAEs and BPA does not cause adverse health effects, as HQ and HQ-AA values were found to be less than 1.0. According to the HQ and HQ-AA values, the risk of PAE congeners were found to be in order of DEHP > DBP, BBP, DEP and DEHP > DBP, BPA, BBP for winter season, whereas in summer the risk order is DEHP > DBP, DEP and DEHP > BPA, DBP respectively in the studied area. This suggests that seasonal variation has a more significant impact on high-risk phthalates than low-risk species which was also supported by the findings of Luo et al*.*^23^. However, other co-occurring contaminants, chemicals employed during water treatment, and combinatorial effect of these, may also alter the HQ value.

**Table 2:** Human exposures and risk assessment of PAEs and BPA through drinking water.

Analytes	EDI (µg/kg bw/day)	HQ (RfD)	HQ (RfD-AA)	Contribution via drinking water	Excess cancer risk
**Winter**					
DMP	2.662	–	–	–	–
DEP	0.193	0.0002	–	–	–
DBP	33.450	0.334	0.334	334.496	–
BBP	0.338	0.002	0.001	0.068	–
DEHP	702.750	35.137	23.425	1405.500	9.838
DNOP	0.358	–	–	–	–
BPA	0.096	–	0.008	–	–
**Summer**					
DMP	0.046	–	–	–	–
DEP	0.012	1.51875E−05	–	–	–
DBP	1.106	0.011	0.011	11.057	–
BBP	–	–	–	–	–
DEHP	73.276	3.664	2.443	146.552	1.026
DNOP	0.030	–	–	–	–
BPA	0.192	–	0.015	–	–

### Strengths and limitations

This study is the first to report the priority pollutants such as PAE congeners and BPA in the drinking water system of India. This study has analyzed the seasonal variation to better understand the influence of temperature on the abundance and distribution of these EDCs in the drinking water system. These findings will provide the reference value of phthalates and BPA in the Indian drinking water system for future studies. Additionally, the exposure level of phthalates and BPA through consumption of drinking water was determined and the associated health risk in the population estimated to provide an overview and magnitude of the problem.

This study has some limitations, long term exposure of PAEs and BPA on the individuals was not assessed. Moreover, larger sample size could give us better understanding of the seasonal variation of the targeted analytes as well as interpretation of exact health risk associated with such pollutants.

## Conclusions

This study provides an overview of the occurrence and concentrations of PAEs and BPA in the source and drinking water of Jabalpur, India. Our findings imply that temperature variation significantly affects the relative abundance and distribution pattern of targeted EDCs i.e., PAEs and BPA. These EDCs were ubiquitously found throughout the source water and drinking water with high detection values for DEHP, DNOP, and DBP in the study area. Source water samples containing high levels of phthalates reflect intensive anthropogenic activities (industrial and agricultural practices) and abiotic factors. Moreover, it also highlights the inefficiency of WTP in managing these emerging pollutants in developing countries like India. DEHP in source and drinking water surpassed the threshold value, thus posing a significant risk to human health.

The carcinogenic risk for DMP, DEP, DBP, BBP, DNOP were lower than the upper acceptable carcinogenic risk level (10^−4^), while the DEHP’s carcinogenic risk value crossed the acceptable range. Thus, our findings contribute to the information of cancer risk of DEHP through ingestion of drinking water and highlight the risk at community level. Although DMP, DEP, DBP, BBP, and DNOP had non-carcinogenic risk values less than 1, but other routes of exposure might also influence this value. Non-carcinogenic risks induced by DEHP through drinking water ingestion poses significant health risk with consequences such as endocrine, neural, hepatic, nephrotic and cardiac toxicity.

This study emphasizes the importance of adequate phthalate monitoring in India's drinking water system to ascertain the human and environmental health status. Thus, bio-monitoring is needed across the country, especially in areas where anthropogenic activities are high, and industrial waste management policies should be imposed to minimize contamination of hazardous pollutants in drinking water. Concurrently, alternatives of these plasticizers should be developed to substitute such toxic chemicals. Future research should focus on phthalate monoesters (MPAEs) to estimate direct exposure by drinking water. Furthermore, the exposure to these EDCs is of epidemiological significance and need to be tracked even in serum and urine samples that can serve as biomarkers in various bio-monitoring programs.

## Supplementary Information

Supplementary Information.

## Data Availability

The data that support the findings of this study are available on request from the corresponding author.
