# Supplementary material for: Occurrence and seasonal disparity of emerging endocrine disrupting chemicals in a drinking water supply system and associated health risk
Source: Sci Rep. 2022 Jun 3;12:9252. doi: 10.1038/s41598-022-13489-3 (PMC9166704; doi:10.1038/s41598-022-13489-3)
Supplement: Supplementary file 1 — Supplementary Information. [file 41598_2022_13489_MOESM1_ESM.docx]

**Occurrence and seasonal disparity of emerging endocrine disrupting chemicals in a drinking water supply system and associated health risk**

Manoj Kumawat^1^, Poonam Sharma ^1^, Namrata Pal ^1^, MeenuMariya James ^1^, Vinod Verma ^2*^, Rajnarayan R Tiwari ^1^, Swasti Shubham ^1*^, Devojit Kumar Sarma^1*^, Manoj Kumar ^1#*^

^1^ ICMR- National Institute for Research in Environmental Health, Bhopal Bypass Road, Bhouri,

Bhopal - 462030, Madhya Pradesh, India

^2^ Stem Cell Research Centre, Department of Hematology, Sanjay Gandhi Post-Graduate Institute of Medical Sciences, Lucknow - 226014, Uttar Pradesh, India

**# Corresponding author**

Manoj Kumar

ICMR- National Institute for Research in Environmental Health, Bhopal

Bypass Road, Bhouri, Bhopal - 462030, Madhya Pradesh, India

Email: manoj15micro@yahoo.co.in

* Equal contribution

**SUPPLEMENTARY FILE**

**Supplementary Table:1Area-wise detection frequency (Df) of phthalates and BPA in drinking water samples (where *N*= Total no. samples, and *n* = no. of samples in which PAE and BPA detected).**

| **ANALYTES** | **AREA WISE DETECTION FREQUENCY** | | | | | | | | | | | | |
| --- | --- | --- | --- | --- | --- | --- | --- | --- | --- | --- | --- | --- | --- |
|  | **WINTER** | | | | | | | | | | | | |
|  | **BHONGDWAR** | | | **RANJHI** | | | **LALPUR** | | | **RAMNAGRA** | | | |
|  | **n** | **N** | **%** | **n** | **N** | **%** | **n** | **N** | **%** | **n** | **N** | **%** |  |
| BPA | 22 | 23 | 95.65 | 19 | 25 | 76 | 25 | 25 | 100 | 22 | 23 | 95.65 |  |
| DMP | 23 | 23 | 100 | 25 | 25 | 100 | 25 | 25 | 100 | 23 | 23 | 100 |  |
| DEP | 21 | 23 | 91.30 | 15 | 25 | 60 | 21 | 25 | 84 | 23 | 23 | 100 |  |
| DBP | 23 | 23 | 100 | 25 | 25 | 100 | 25 | 25 | 100 | 23 | 23 | 100 |  |
| BBP | 12 | 23 | 52.17 | 9 | 25 | 36 | 16 | 25 | 64 | 11 | 23 | 47.83 |  |
| DEHP | 23 | 23 | 100 | 25 | 25 | 100 | 25 | 25 | 100 | 23 | 23 | 100 |  |
| DNOP | 14 | 23 | 60.87 | 6 | 25 | 24 | 16 | 25 | 64 | 21 | 23 | 91.30 |  |
| **ANALYTES** | **SUMMER** | | | | | | | | | | | | |
|  | **BHONGDWAR** | | | **RANJHI** | | | **LALPUR** | | | **RAMNAGRA** | | | |
|  | **n** | **N** | **%** | **n** | **N** | **%** | **n** | **N** | **%** | **n** | **N** | **%** |  |
| BPA | 24 | 24 | 100 | 25 | 25 | 100 | 25 | 25 | 100 | 23 | 23 | 100 |  |
| DMP | 3 | 24 | 12.5 | 3 | 25 | 12 | 20 | 25 | 80 | 4 | 23 | 17.39 |  |
| DEP | 24 | 24 | 100 | 25 | 25 | 100 | 25 | 25 | 100 | 23 | 23 | 100 |  |
| DBP | 24 | 24 | 100 | 25 | 25 | 100 | 25 | 25 | 100 | 23 | 23 | 100 |  |
| BBP | - | - | - | - | - | - | - | - | - | - | - | - |  |
| DEHP | 22 | 24 | 91.67 | 25 | 25 | 100 | 17 | 25 | 68 | 20 | 23 | 86.96 |  |
| DNOP | 21 | 24 | 87.5 | 24 | 25 | 96 | 25 | 25 | 100 | 23 | 23 | 100 |  |

**Supplementary Table: 2 Area wise concentrations of ∑6PAEs (Dimethyl phthalate (DMP), Diethyl phthalate (DEP), Di(2-ethylhexyl) phthalate (DEHP), Benzyl butyl phthalate (BBP), Di-n-butyl phthalate (DBP), and Di-n-octyl phthalate (DNOP) in water samples collected from Household Over Head (OH) Tanks and Drinking water (DW) during summer and winter. Results are reported in ug/l.**

**Supplementary Fig. 1Calibration curve of (a) dimethyl phthalate (DMP); (b) diethyl phthalate (DEP); (c) dibutyl phthalate (DBP); (d) benzyl butyl phthalate (BBP); (e) bis (2-ethylhexyl) phthalate (DEHP); (f) di-n-octyl phthalate (DNOP) at a concentration range from 10-900 ppb and; (g) bisphenol A (BPA) at concentration range from 50- 900 ppb.**
